# Analysing multi-perspective patient-related data during laparoscopic gynaecology procedures

**DOI:** 10.1038/s41598-023-28652-7

**Published:** 2023-01-28

**Authors:** Nour Aldeen Jalal, Tamer Abdulbaki Alshirbaji, Bernhard Laufer, Paul D. Docherty, Thomas Neumuth, Knut Moeller

**Affiliations:** 1 Institute of Technical Medicine (ITeM), Furtwangen University, 78054 Villingen-Schwenningen, Germany; 2 Innovation Center Computer Assisted Surgery (ICCAS), University of Leipzig, 04103 Leipzig, Germany; 3 Department of Mechanical Engineering, University of Canterbury, Christchurch, 8041 New Zealand

**Keywords:** Health care, Biomedical engineering

## Abstract

Fusing data from different medical perspectives inside the operating room (OR) sets the stage for developing intelligent context-aware systems. These systems aim to promote better awareness inside the OR by keeping every medical team well informed about the work of other teams and thus mitigate conflicts resulting from different targets. In this research, a descriptive analysis of data collected from anaesthesiology and surgery was performed to investigate the relationships between the intra-abdominal pressure (IAP) and lung mechanics for patients during laparoscopic procedures. Data of nineteen patients who underwent laparoscopic gynaecology were included. Statistical analysis of all subjects showed a strong relationship between the IAP and dynamic lung compliance (r = 0.91). Additionally, the peak airway pressure was also strongly correlated to the IAP in volume-controlled ventilated patients (r = 0.928). Statistical results obtained by this study demonstrate the importance of analysing the relationship between surgical actions and physiological responses. Moreover, these results form the basis for developing medical decision support models, e.g., automatic compensation of IAP effects on lung function.

## Introduction

**Figure 1:**
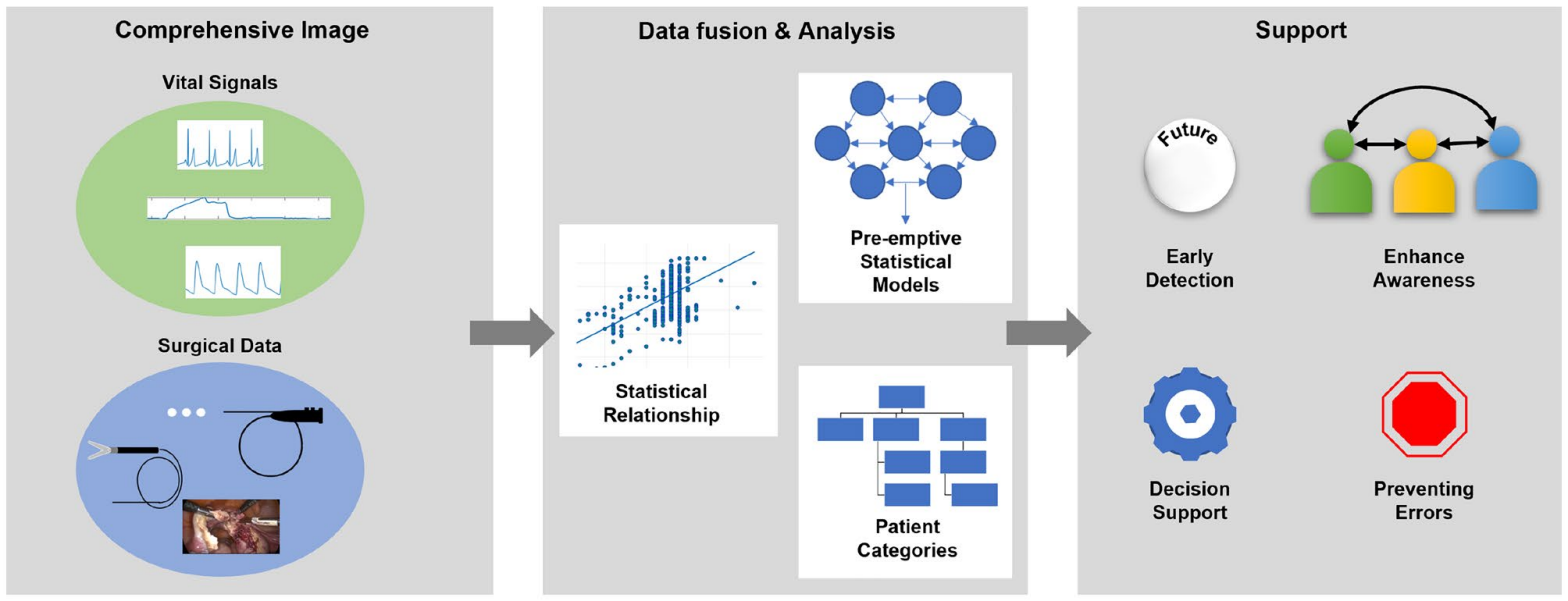
Schematic representation of a data-driven pipeline to establish a context-aware system inside the OR. Comprehensive Image: data from both the surgical and patient sides are combined. Data fusion and analysis techniques are employed to fuse data from different perspectives. Pre-emptive models are then developed to enhance surgical treatment by providing support to the medical staff (surgical and anaesthesiologic teams).

Operating theatres have evolved with advances in medical technology^1^. Future operating rooms (OR) will increase their reliance on intelligent, context-aware systems (CAS). CAS can analyse multiple channels of data available inside the OR to enhance patient safety and efficiency of surgical treatment^1,2^. CAS will enable transformation of surgeries to become more data-driven rather than based on each clinicians’ unique experiences. Thus, the CAS will provide the surgical team and anaesthesiologic team with real-time comprehensive knowledge about the patient status inside the OR without the need for verbal communication across teams. This knowledge is generated by fusing data from different perspectives (such as data from surgery and anaesthesiology) and employing previously established predictive and prescriptive models. In this context, personalised treatment will be enabled, and surgery will thus be performed in a high information environment regardless the experience of the medical teams. Furthermore, surgical complications and medical errors caused by the high complexity inside the OR could potentially be avoided, and a better collaboration and communication between medical teams can be promoted^1–3^ (see Fig. 1).

Recently, rapid developments in data science and artificial intelligence (AI) techniques, particularly deep learning (DL), have boosted active research in the field of computer-assisted intervention (CAI)^1,4,5^. Consequently, Surgical Data Science (SDS) was introduced as a scientific discipline that aims at “improving the quality of interventional healthcare and its value through capture, organisation, analysis, and modelling of data”^1^. Previous work proposed various approaches and methodologies to establish CAS components that meet the goals of SDS. Indeed, most published approaches advance one of three goals within SDS: Firstly, a target application was addressed, such as recognising surgical activities^6–11^, detecting surgical tools^12–15^, or predicting remaining time of surgery^16–18^. Secondly, specific data sources were chosen and utilised as an input, such as laparoscopic video^8,19,20^, or sensor-based data^21,22^. Finally, an appropriate method was developed to achieve the target defined in the paper.

The main drawback that hindered the SDS evolution is the lack of labelled, comprehensive, and well-representative data. This is mainly due to the current technical infrastructure inside the OR, that does not facilitate data acquisition from the variety of available devices. Additionally, data interoperability is still not supported between medical devices of different manufactures. Therefore, several recent initiatives focused on leveraging the interoperability between medical devices inside the OR. The research project OR.NET^23^ paved the way for better medical device networking by establishing the IEEE 11073 Service-oriented Device Connectivity (SDC) standard. SDC standard enables vendor-independent data communication and exchanging between medical devices. However, the approved SDC standard so far represents the core part, and continuous research is still, therefore, required to develop high-level standards for networking specific categories (like the German PoCSpec project^24^, funded by the Federal Ministry of Economic Affairs and Energy). InnOPlan project^25,26^ introduced a smart OR device platform, based on established standards like SDC, that focuses on combining relevant data of medical devices to enhance efficiency and safety inside the OR.

Only a few relatively small, and single-perspective (i.e., contains surgical data) datasets are publicly accessible to researcher. Primarily, laparoscopic videos have been the dominant data used in literature, especially for surgical workflow analysis. This can be interpreted by the nature of laparoscopic surgeries, which provide an easily accessible source of surgical video data. The Cholec80^8^ and EndoVis^19,20^ are the most widely known laparoscopic-video datasets utilised by SDS researchers for surgical phase recognition and surgical tool presence detection. The EndoVis dataset contains, besides the laparoscopic video, medical data of some surgical devices (e.g., Insufflator, light, laparoscopic camera). However, the analysis of physiological data (data from anaesthesiology) has also shown potential to improve patient safety during the surgery and postoperative outcome^27^. In particular, machine learning techniques were applied on anaesthesiology data in order to predict occurrence of intraoperative events such as hypotension^28,29^, or hypoxaemia^30^, control the delivery of anaesthetic agent^31^, or estimate the depth of anaesthesia^32^. Hatib et al. employed a logistic regression model to predict hypotension up to 15 minutes in patients using arterial pressure signal^28^. Lundberg et al. developed an explainable machine learning method to predict the occurrence of hypoxaemia^30^. The method utilised real-time data from the anaesthesiology and patient monitor (such as arterial blood oxygen saturation (SPO$_{2}$) and tidal volume) to predict hypoxaemia in the next 5 min.

Despite the great potential of current SDS approaches, it is worth noting that, these approaches were conducted using a single-perspective data. Furthermore, studies that evaluate the relationship between surgical actions and corresponding changes in the physiological parameters of the patient are still lacking. Therefore, future studies should focus on fusing the heterogeneous data (video, respiratory, pulse oximeter) available inside the OR to generate a comprehensive description of the overall status. Specifically, fusing physiological data (anaesthesiology side) with surgical data (surgery side) is necessary for developing CAS medical decision support models.

In this paper, a data fusion approach was evaluated on physiological data (anaesthesiologist side) and surgical data (surgeon side) acquired during gynaecological laparoscopic procedures. Data from surgical and anaesthesiologic devices was first collected and pre-processed to generate a dataset of physiological and surgical data. The relationship between the intra-abdominal pressure and lung mechanics of the patient was evaluated, and a statistical correlation coefficient was computed.

The novel contributions of this paper are: (1) A real-time continuous data recording system that facilitates synchronous data collection from surgical devices, anaesthesia machine and patient monitor. (2) Synthesis of a unique dataset composed of synchronised heterogeneous multi-modal data acquired during laparoscopic gynaecology procedures. The data is composed of laparoscopic videos, medical device data (e.g., intra-abdominal pressure (IAP) signal), mechanical ventilation signals (e.g., airway pressure), and vital signs of the patient (e.g., ECG signal). (3) A descriptive data analysis was performed to reveal patient status changes during gynaecological procedures. The potential benefits of this approach are exemplarily demonstrated by revealing the clinically relevant interaction between intra-abdominal pressure changes and lung mechanics.

## Results

**Figure 2:**
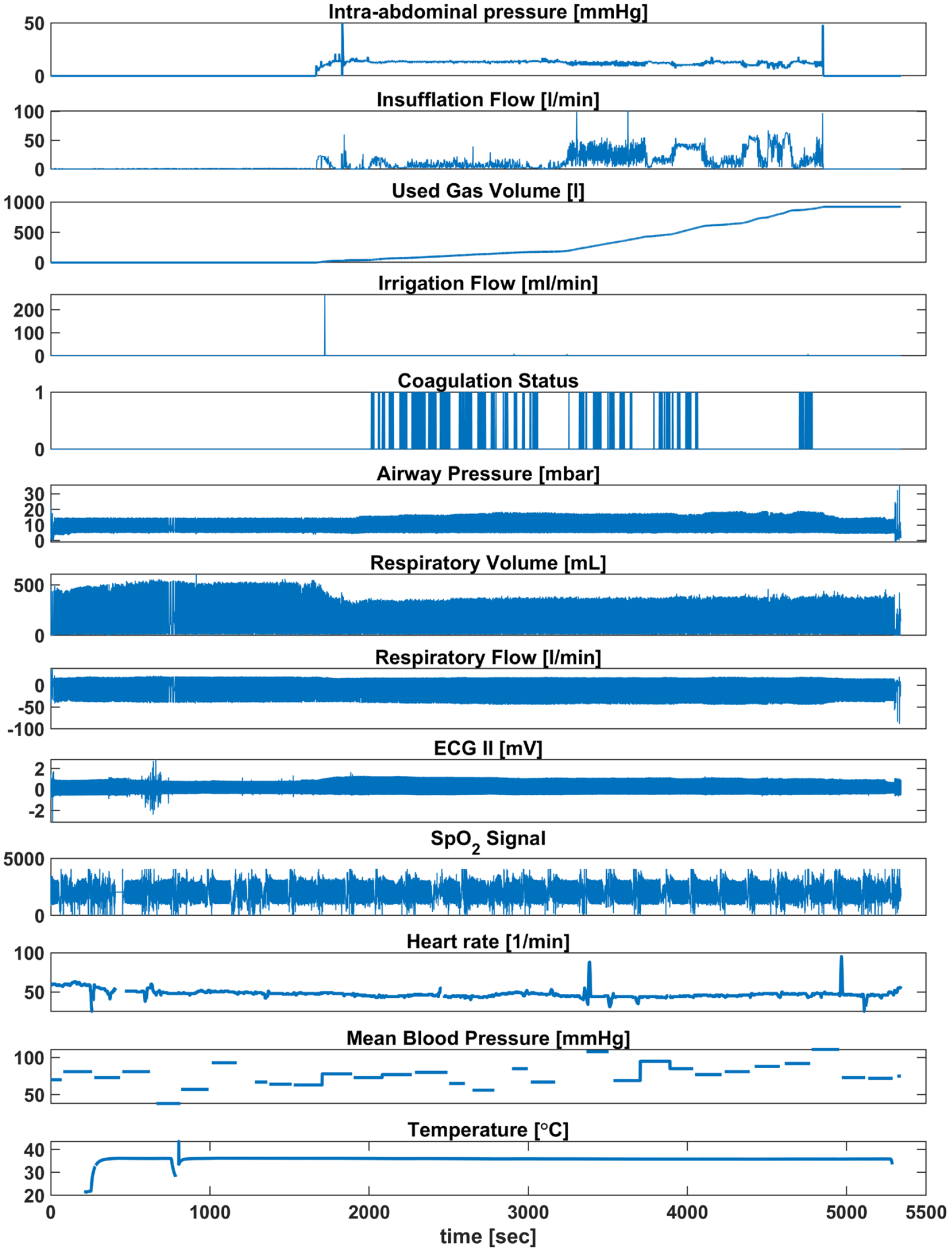
Visualisation of collected signals from included medical devices. The top five graphs display surgical data, such as intra-abdominal pressure.

**Figure 3:**
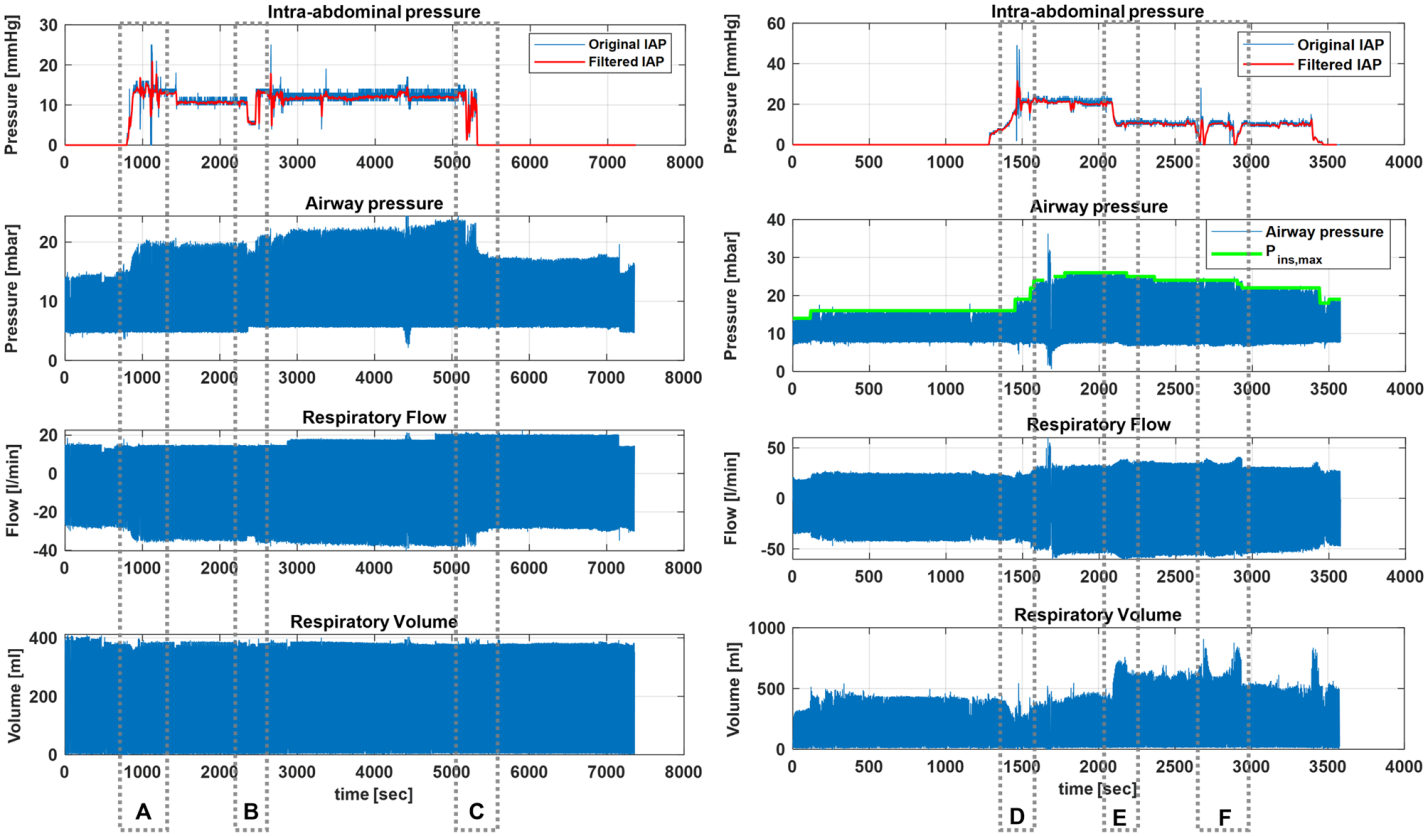
IAP, airway pressure, respiratory flow, and volume of VCV-patient (Subject 2, left) and PCV-patient (Subject 15, right). A, B, C show effects of changing IAP on respiratory parameters of VCV-patient, where *PIP* and negative peak flow increase or decrease when IAP increases or decreases, respectively. Similarly, D, E, F show effects of IAP in PCV-patient. Here, ${V_{T}}$ changes according to changes in IAP.

A full example of collected medical data from patient monitor, anaesthesia machine and surgical devices is presented in Fig. 2. These data represent time-series signals recorded at different sampling frequencies. In addition, laparoscopic videos, device settings, and alarms were also acquired. Figure 3 shows IAP, airway pressure, respiratory flow, and respiratory volume of a volume-controlled ventilated patient (VCV-patient) and a pressure-controlled ventilated patient (PCV-patient).

**Figure 4:**
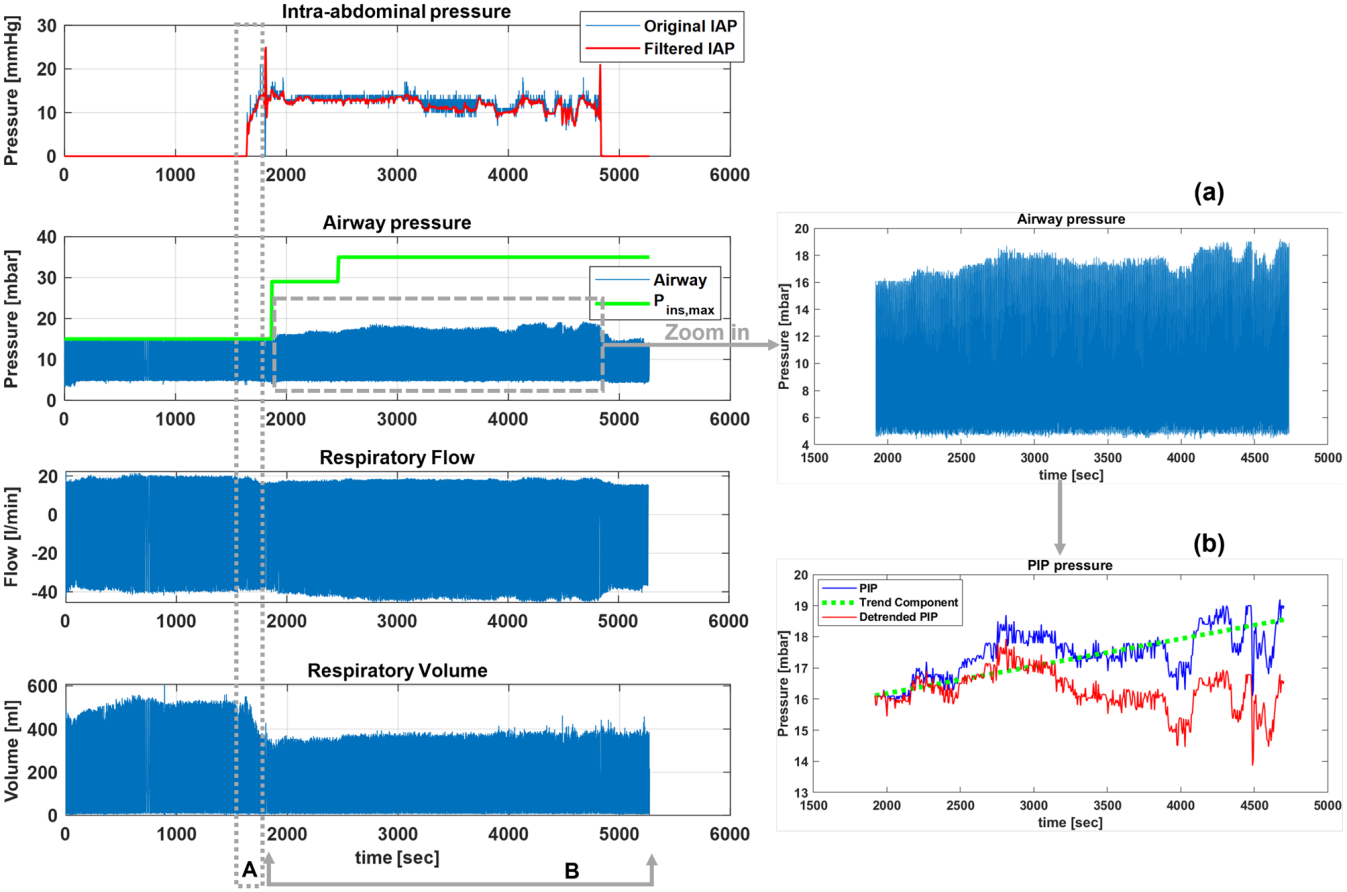
IAP, airway pressure, respiratory flow, and volume of PCV-patient (Subject 12). (A) *PIP* reached ${P_{ins,max}}$, and increasing IAP, therefore, caused a drop in the inspirational tidal volume. (B) *PIP* could not reach ${P_{ins,max}}$, and changes in IAP, therefore, affected *PIP*. (**a**) Zoom in of airway pressure, (**b**) Extracted *PIP*, a trend component, and detrended *PIP*.

Typically, changing intra-abdominal pressure (IAP) results in changes in peak airway pressure (*PIP*) or tidal volume (${V_{T}}$) in volume-controlled ventilated or pressure-controlled ventilated patients, respectively. This can be observed for Subject 2 (VCV-patient) and Subject 15 (PCV-patient) in Fig. 3. In contrast, changes in *PIP* and ${V_{T}}$ were observed for another PCV-patient (Subject 12) when IAP changed (Fig. 4). More precisely, when *PIP* reached the pre-set inspirational pressure (${P_{ins,max}}$), increasing IAP resulted in a drop in the ${V_{T}}$ (Fig. 4A). Inversely, the *PIP* changed in accordance with the IAP when the *PIP* was lower than ${P_{ins,max}}$ (Fig. 4B). Figure 4b also shows a linear trend component of the *PIP* of subject 12.

**Figure 5:**
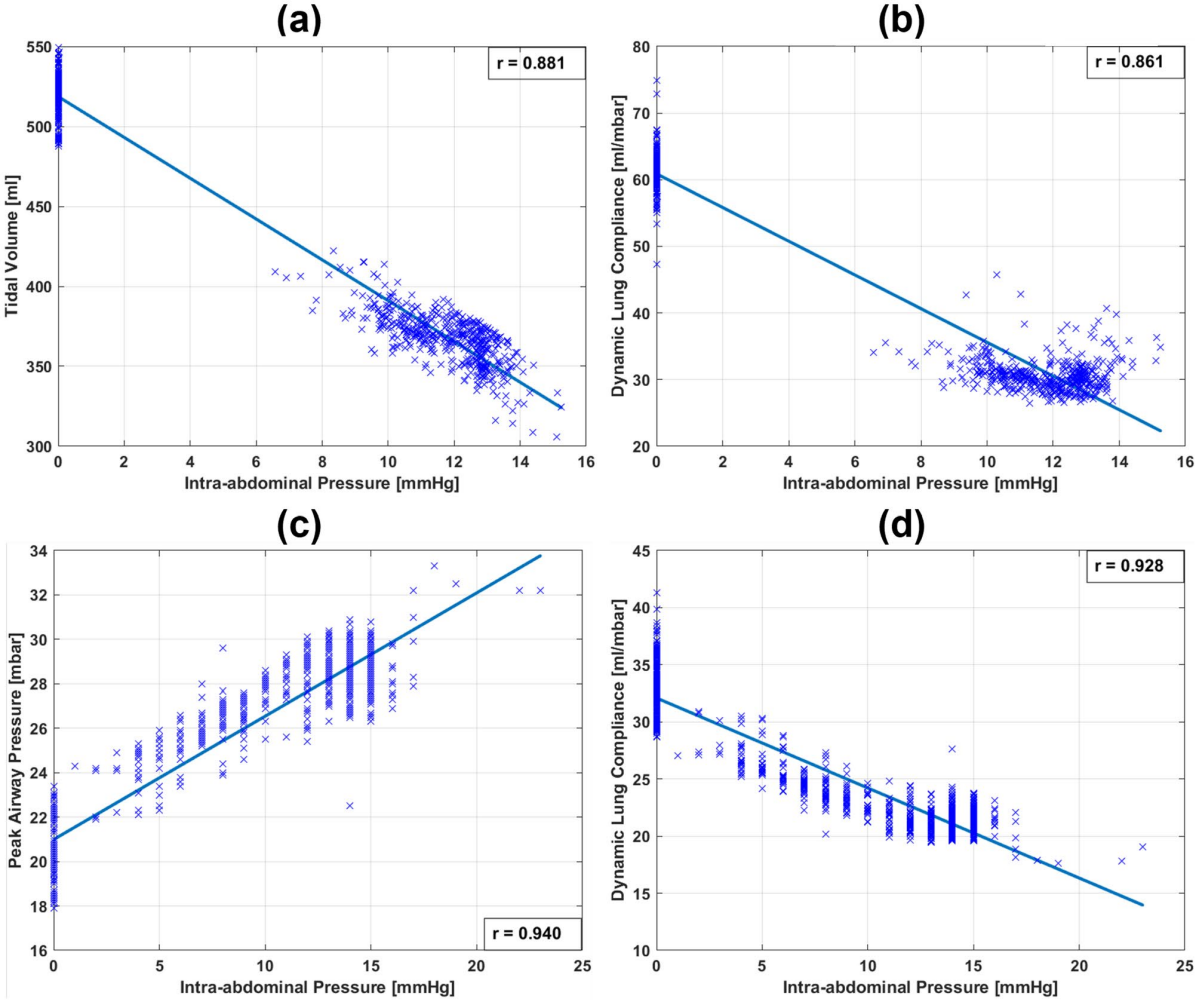
Linear regression correlation for two subjects. (**a**,**b**) are the linear correlations between tidal volume and dynamic lung compliance with IAP, respectively, for a PCV-patient (Subject 12). (**c**,**d**) are the linear correlations between peak airway pressure and dynamic lung compliance with IAP, respectively, for a VCV-patient (Subject 1).

**Figure 6:**
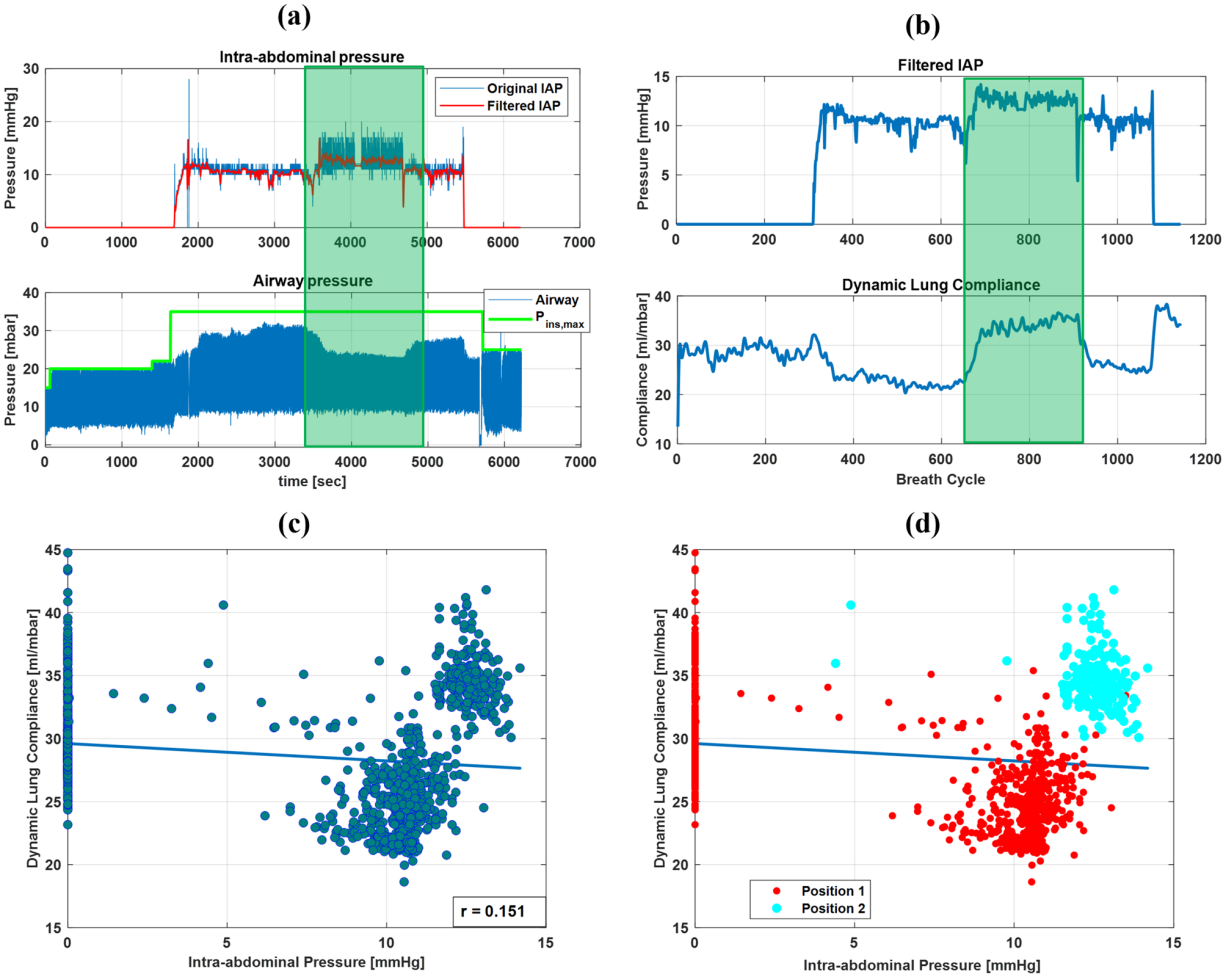
Patient repositioning effect on lung compliance. (**a**) IAP and airway pressure of Subject 11 during the surgery. (**b**) Dynamic lung compliance and IAP values extracted for breath cycles. The green area in (**a**,**b**) represent a repositioning of the patient during the procedure. It shows a drop in *PIP* and increase in ${C_{dyn}}$ while the IAP was almost constant, and all ventilation settings were constant. (**c**) Linear regression correlation between ${C_{dyn}}$ and IAP where r value was very small. (**d**) a scatter of IAP and ${C_{dyn}}$ grouped by patient positioning.

Pearson’s correlation coefficients (r) for the relationships between the intra-abdominal pressure (IAP) and the peak airway pressure (*PIP*) and the dynamic lung compliance $({C_{dyn}})$ of VCV-patients are presented in Table 1 (S1-S8). Additionally, Pearson’s correlation coefficients for PCV- patients are listed in Table 1 (S9-S19). Table 2 presents a summary of the linear correlation results for all subjects. The regression relationships between the IAP and ${C_{dyn}}$, *PIP*, or ${V_{T}}$ for Subject 1 and Subject 12 are presented in Fig. 5. To highlight the effects of patient positioning/repositioning during the surgery, real-time recordings of the IAP and airway pressure of Subject 11 (PCV-patient) along the surgical procedure are presented in Fig. 6. The breath-by-breath ${C_{dyn}}$ and the corresponding IAP are also presented in Fig. 6a. Additionally, a scatter of the IAP and the ${C_{dyn}}$ grouped by patient positioning is presented in Fig. 6d. Since patient 11 was shifted during surgery - their data represents an aberration from the surgical protocol and their data will not be considered in correlation. However, their case represents an important consideration for SDS and thus will be presented and discussed in isolation.

**Table 1:** Pearson’s correlation coefficients (r) between the IAP and lung mechanics values for all subjects.

Subject	Pearson correlation coefficient (r)					
	$\hbox {C}_{\textrm{dyn}}$ versus IAP		PIP versus IAP		$\hbox {V}_{\textrm{T}}$ versus IAP	
	C	MLR	C	MLR	C	MLR
1	0.928	0.944	0.940	0.961	–	–
2	0.891	0.967	0.857	0.978	–	–
3	0.736	0.914	0.736	0.917	–	–
4	0.913	0.956	0.921	0.992	–	–
5	0.688	0.792	0.803	0.813	–	–
6	0.901	0.953	0.942	0.971	–	–
7	0.842	0.893	0.820	0.930	–	–
8	0.831	0.872	0.823	0.872	–	–
9	0.882	0.901	0.854	0.912	0.150	0.823
10	0.686	0.841	0.683	0.881	0.110	0.253
11	0.460	0.484	0.675	0.787	0.151	0.848
12	0.861	0.981	0.790	0.873	0.881	0.921
13	0.764	0.940	0.723	0.991	0.210	0.751
14	0.931	0.952	0.840	0.992	0.150	0.770
15	0.921	0.941	0.770	0.991	0.111	0.900
16	0.855	0.895	0.693	0.968	0.136	0.691
17	0.777	0.898	0.741	0.972	0.294	0.670
18	0.706	0.850	0.796	0.980	0.395	0.900
19	0.702	0.885	0.882	0.988	0.060	0.767

*C* is the linear regression correlation between the IAP and the *PIP*, the ${C_{dyn}}$ and the $V_{T}$. *MLR* is the multiple/multivariate linear regression correlation after including ventilation settings.

**Table 2:** Summary of linear correlation results.

Subject	Pearson correlation coefficient (r)					
	$\hbox {C}_{\textrm{dyn}}$ versus IAP		PIP versus IAP		$\hbox {V}_{\textrm{T}}$ versus IAP	
	C	MLR	C	MLR	C	MLR
All Subjects	0.805	0.887	0.804	0.934	–	–
Subject 11 excluded	0.824	0.910	0.811	0.942	–	–
VCV-subjects	0.841	0.910	0.854	0.928	–	–
PCV-subjects & Subject 11 excluded	0.811	0.908	0.777	0.954	0.252	0.735

The listed values represent mean of Pearson’s correlation coefficients (r), where IAP is intra-abdominal pressure, ${C_{dyn}}$ is dynamic lung compliance, *PIP* is peak airway pressure, and ${V_{T}}$ is tidal volume.

## Discussion

In this study, a data recording system that facilitates recording of data from multi-vendor medical devices inside the OR was presented. This system enables acquisition of a unique combination of medical surgical data and patient-related information intraoperatively and storing them in a structured manner. Exemplarily for potential uses, a descriptive analysis was carried out to establish the relationships between the surgical actions taken by the surgeon and changes on physiological parameters of the patient, here the respiratory systems’ mechanics. In this context, correlations between the intra-abdominal pressure and lung mechanics of patients undergoing laparoscopic procedures were investigated.

### Data recorder

The developed data recording system was tailored for a specific hospital (Schwarzwald-Baar clinics in Villingen-Schwenningen, Germany) and a specific type of surgical procedure (i.e., laparoscopy). Therefore, it is worth discussing what parts of the system are specific to this hospital and what can be suitable for other hospitals. First, data acquisition was performed in the integrated operating room OR1 FUSION (provided by KARL STORZ SE & Co.KG, Tuttlingen, Germany). Therefore, acquiring data from surgical devices relied on coupling the surgical devices via the STORZ communication bus (SCB). Second, the patient monitor and anaesthesia machine were provided by Philips GmbH and Löwenstein Medical GmbH & Co. KG, respectively. In typical OR setups, a standard for data exchange does not exist. Hence, software for data acquisition were developed using the data communication protocol provided for each device by its manufacture. Consequently, developed software can be used in other hospitals when the same medical devices are used. The hardware components (i.e., active and passive converters) that were utilised to couple the medical devices with the computer are specific to the infrastructure in the OR. These components were evaluated before actual recording and checked in terms of signal distortion and data loss. Moreover, active converters (e.g., ECB-SCB converter used to connect the electrosurgical unit (provided by ERBE Elektromedizin GmbH) to STORZ devices) are class IIB medical devices, and they were outside the sterilised area without any impact on the workflow.

### Data analysis

Increasing the IAP during the laparoscopic procedures impacts the respiratory system of the patient, where the IAP acts in the reverse direction of the ventilation-driving pressure. Therefore, the effects of the IAP on respiratory system parameters were investigated. Statistical analysis showed a strong relationship between the IAP and lung mechanics for almost all patients during laparoscopy. When all subjects were considered, the mean Pearson’s correlation coefficient (r) of 0.887 for the multiple linear regression (*MLR*) indicates a strong linear relationship between the IAP and the ${C_{dyn}}$ (Table 2). The same strong correlations can be seen for both VCV-patient and PCV-patient with mean r values of 0.910 and 0.908, respectively (see Table 2). Moreover, altering the settings of ventilation during surgery affects respiration parameter of the patient. Hence, considering these changes in settings is essential to get the exact correlation with the IAP. In this context, multiple linear regression models were further analysed. As can be seen from Tables 1 and 2, the correlation coefficients for almost all subjects obtained for *MLR* improved by a large margin over the *C* correlation.

In VCV-patients, increasing the IAP at the start of the procedure caused an immediate increase in *PIP* to maintain the target tidal volume $({V_{T,target}})$ (Fig. 3A). Additionally, changes in IAP between abdomen insufflation and deflation correlated positively with the *PIP* (Fig. 3B). After abdomen deflation, the *PIP* dropped back to normal in accordance due to manual changes of ventilation settings by the anaesthesiologist. Similarly, the same patterns can be observed on the respiratory flow curve, where the peak negative flow showed similar changes as the *PIP*. Conversely, the ${V_{T}}$ did not get affected because the volume-controlled mode ensured patients received the pre-set ${V_{T,target}}$.

In PCV, the ventilator focuses on regulating pressure during mechanical ventilation. Therefore, the increased IAP after insufflating the abdomen cavity with CO$_{2}$ caused a drop in the tidal volume delivered in all subjects except Subjects 9, 10, 11 and 12. Figure 3 (right) shows the IAP and the respiratory signals (airway pressure, respiratory volume, and respiratory flow) for Subject 15. As can be seen, there was no relationship between the IAP and the *PIP*, whereas changing the IAP affected the tidal volume delivered. In other words, decreasing or increasing the IAP resulted in an increase or decrease in the tidal volume, respectively (see Fig. 3D,E).

It is worth noting that for PCV patients, the *PIP* pressure generally reached the pre-set inspiration pressure (${P_{ins,max}}$) during the duration of the procedure. Interestingly, different responses were observed for some PCV-subjects (Subject 9, 10, 11 and 12). In particular, the *PIP* of these subjects had a relationship with the changes in the IAP even in PCV. For instance, Fig. 4 shows real-time data of Subject 12. The first period (A) is the period when the abdomen was insufflated, while the second period (B) is the period after the ${P_{ins,max}}$ was increased by the anaesthesiologist until the abdomen deflation. The main characteristic of these two sections (A, B) was the differences between the *PIP* and ${P_{ins,max}}$. In Fig. 4A, the *PIP* was equivalent to the ${P_{ins,max}}$, and the ${V_{T}}$, expectedly, dropped immediately after the IAP was increased, where the *PIP* was not affected. Inversely, Fig. 4B shows changes in the *PIP* during elevated IAP, with no concomitant change in ${V_{T}}$. Here, the ${P_{ins,max}}$ was increased by the anaesthesiologist by 14 mbar and subsequently even more by 6 mbar. However, the *PIP* never reached the ${P_{ins,max}}$. Additionally, the *PIP* had a linear trend component (see Fig. 4a,b). Thus, to get an accurate correlation, the trend component was removed from the *PIP* prior to analysing the statistical relationship for those subjects. Ultimately, increasing the IAP resulted in an apparent decrease in the ${C_{dyn}}$ of all patients (PCV- and VCV-patients), and vice-versa. This trend in the lung compliance can be seen in Fig. 5b,d. This reduction in apparent lung compliance during periods of elevated IAP produced an increase in the *PIP* in VCV-patients, and a decrease in the tidal volume in PCV-subjects.

In PCV-patients, changes in ${V_{T}}$ and IAP do not seem to be closely related (r = 0.25) (see Tables 1 and 2). However, subject 12 exhibited a comparatively high correlation (r = 0.881). Whereas the mean r for the relationship between the IAP and the ${C_{dyn}}$ and *PIP* were 0.811 and 0.777, respectively. For Subjects 9-12, low r values for the ${V_{T}}$-IAP correlation were due predominantly to the consistency of ${V_{T}}$ during surgery. For the Subjects 13–19, ${V_{T}}$ was highly correlated to the ${P_{ins,max}}$ that was altered many times during the surgical procedure. Therefore, considering the ${P_{ins,max}}$ (as well as other settings) when calculating the correlation is important. This can be seen by comparing the r values of *C* and *MLR* for all PCV-subjects. In a similar way, the high r values obtained by *MLR* for the relationship between IAP and *PIP* for PCV-subjects does not always express a high correlation between them, but these high r values resulted from the relationship between the *PIP* and ${P_{ins,max}}$ for Subjects 9-12.

Pearson’s correlation coefficient of 0.484 for Subject 11 indicates a poor linear relationship between the IAP and ${C_{dyn}}$ even when ventilation settings were incorporated. Figure 6a,b show a drop in the *PIP* and increase in ${C_{dyn}}$ even though the IAP was almost constant. The data of this subject was, therefore, retrospectively analysed in light of information from the anaesthesiology protocol and the laparoscopic video. The reason behind this increase in the lung compliance was the repositioning of the patient during the surgery. In fact, this patient was repositioned from the lithotomy to the supine position and then again back to the lithotomy. Several studies have already investigated the effect of patient positioning on lung mechanics during surgeries^33^. However, this study did not intend to capture the effects of varying patient positioning, and thus, the patient yielded aberrant information that was isolated from the grouped statistics.

This study has several limitations. The developed data recording system did not enable coupling all available devices inside the OR (for example, the OR table and infusion pumps were not linked). This limitation was caused by the lack of standardisation in the interoperability of the current clinical setup, and connecting some OR devices to the data recorder was, thus, impossible without disturbing the surgical workflow. Therefore, some important data that might affect the performed analytic study were missing. For instance, patient positioning that can be acquired from the OR table, or fluids introduced to the patient using infusion pumps. Only nineteen female patients were included in this study, and while this is sufficient to determine important trends ($\hbox {p}<0.001$ for all cases), greater patient numbers are required to fully characterise the inter-patient variability in these trends and contribute to confident SDS approaches. It is also possible that patients with abnormal physiology may respond quite differently to those tested. Hence, further research across surgical procedures or patients with different physiology may lead to identification of different outcomes and thus further analysis is required.

However, the presented study has potential to enhance surgical treatment and realise smart ORs. In particular, the statistical relationships between intra-abdominal pressure and lung mechanics shown in Tables 1 and 2 will enhance medical decision support models. It was observed that ${P_{ins,max}}$ was increased by the anaesthesiologist slightly before or after insufflating the abdominal cavity in all PCV-patients to compensate the drop in the tidal volume caused by the IAP-related drop in lung compliance. Furthermore, patients were able to be classified into different categories based on the relationship between the IAP and ${C_{dyn}}$. As a result, individualised support models could be developed to automate the process of compensating the drop in lung compliance by specifying the optimal increase in the ${P_{ins,max}}$ required. This would reduce the burden on the anaesthetist. The statistical results have the potential to characterise lung type for further treatment in the intensive care unit (ICU), and possible instabilities in cardiovascular system or ventilation can be predicted. Additionally, the process of generating the anaesthesiologic protocol is often ad hoc and based on the anaesthesiologist’s experience. By fusing data from surgery and anaesthesiology inside the OR, these protocols can be automatically generated to achieve optimal and consistent patient outcomes. Furthermore, the unique dataset used in this analysis creates a great opportunity to analyse the effects of other surgical actions. For instance, the relationship between electrosurgical activities and the physiological parameters of the patient could be also investigated.

The novel understanding of the interaction between IAP and respiratory mechanics provided by this paper may enable development of novel decision support protocols. However such protocols must be optimised and validated prior to implementation in clinical settings.

## Methods

**Figure 7:**
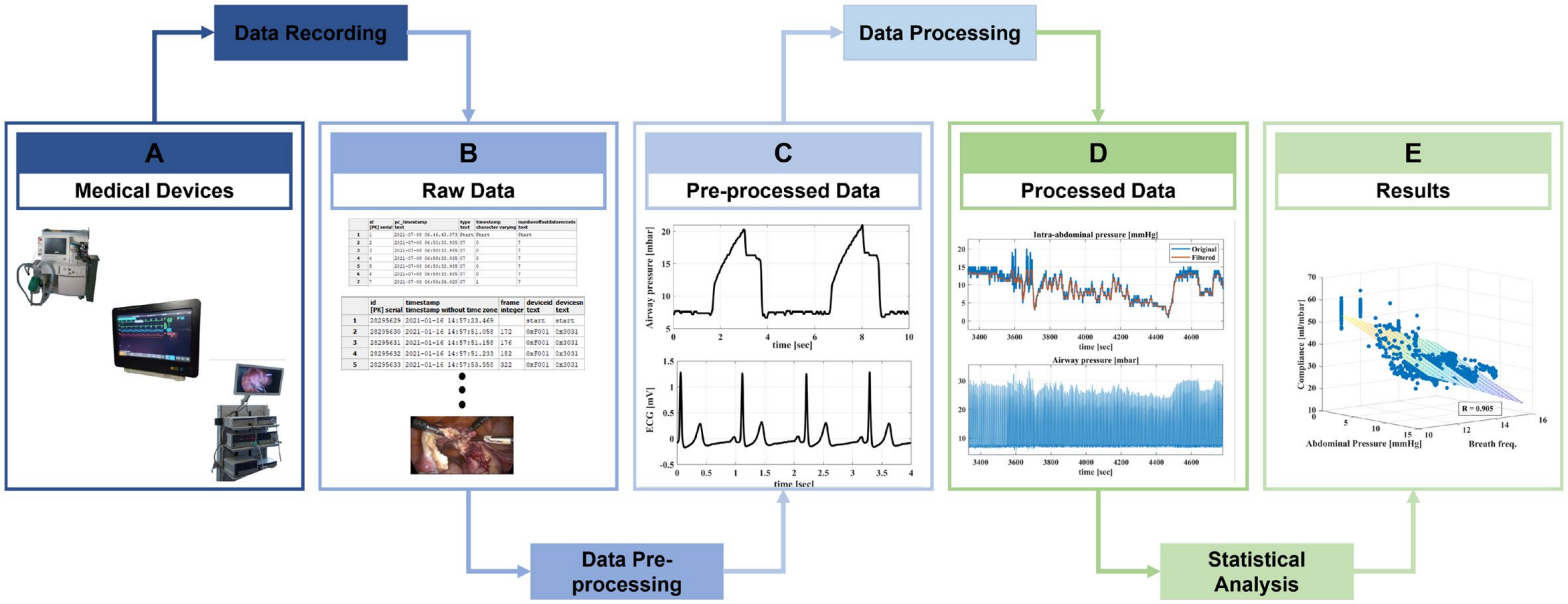
The complete pipeline of the performed study. Data recording: data was collected from the medical devices during laparoscopic gynaecology procedures. In this context, the data sources included the patient monitor, anaesthesia machine, and surgical devices (insufflator, irrigation pump, motor system, electrosurgical unit, and laparoscopic camera). Raw data was stored in tables within a database, and laparoscopic videos were saved as mp4 files. Data pre-processing represents checking the data in term of correctness and completeness, synchronising data from different sources, and cropping all data to the same start and end. Pre-processed data serves as the basis for data analysis and interpretation. Data processing: pre-processed data is processed in a target-oriented manner to provide consistency to the data that is analysed in the following step (e.g., filtering IAP signal). Statistical analysis: statistical relationships between surgical data and vital signs of the patient are analysed.

Figure 7 presents the procedure of the study. The first step was designing a data recording system that facilitates collecting data from surgical devices, the anaesthesia machine and patient monitor during laparoscopic procedures. Acquired raw data was pre-processed, checked for complete and correct data transmission and storage, and saved into readable file formats with a precise timestamp. Then, the data was processed and analysed to address certain clinical goals.

### Data collection

#### Data sources

The developed data recording system enabled synchronous recording of data from surgical devices and patient-status monitoring devices. The surgical devices included the insufflator, irrigation/suction pump, surgical motor system, light source, electrosurgical unit, and the laparoscopic camera. The patient-status monitoring devices included the anaesthesia machine and the patient monitor. The model and manufacture of each device are listed in Table 3.

**Table 3:** Medical devices included in this study and their models and manufacturers.

Nr.	Device	Model	Manufacture
1.	Laparoscopic camera	Image 1	STORZ
2.	Light source	Xenon300	
3.	Insufflator	Electronic endoflator	
4.	Suction/Irrigation pump	Hamou endomat	
5.	Motor system	Unidrive GYN and III	
6.	Electrosurgical unit	VIO 300 D	ERBE GmbH
7.	Anaesthesia machine	LeonPlus neo	Heinen Löwenstein
8.	Patient monitor	Mx800	Philips

The data collected from the surgical devices contained information about the device activation status (On/Off), settings parameters (e.g., target insufflation pressure, target irrigation flow), and actual values (e.g., actual insufflation pressure, actual irrigation/suction pressure). Data acquired from the anaesthesia machine included real-time waves of respiratory and anaesthetic gases (e.g., airway pressure, volume, N$_{2}$O), current values of device and ventilator settings, and active alarms. Similarly, data from the patient monitor included real-time waves (e.g., ECG, SPO$_{2}$) and numeric values (e.g., Blood pressure, temperature). Data sampling frequency was device dependent (Table 4).

**Table 4:** Devices and their data streams acquired during the surgery.

Device	Parameters	Data Type	Sampling rate
Anaesthesia machine	Current values of device settings and ventilation settings (e.g., ventilation mode, pre-set values of inspirational tidal volume, positive end expiratory pressure (PEEP), etc.)	Numerical and String	60 s
	Current values of device state and current ventilation (e.g., actual tidal volume)	Numerical	10 s
	Active alarms	String	When available
	Real-time data streams of airway pressure, flow, volume, CO2, O2, N2O and anaesthetic agent	Wave	20 ms
Patient monitor	Current technical and patient alarms	String	When available
	heart rate, body temperature, blood pressures, oxygen saturation, perfusion indicator	Numerical	1024 ms
	ECG, SPO2	Wave	ECG: 2 ms; SPO2: 8 ms
Laparoscopic camera	Laparoscopic video	Video	40 ms (25 fps)
Electrosurgical unit	Cutting and coagulation signals (active/inactive)	Wave/ binary	40 ms
Insufflator	Target and actual intra-abdominal pressures, target and actual gas flows, supply pressure, utilised gas volume	Wave	40 ms
Irrigation/suction pump	Target and actual irrigation flows, target and actual irrigation pressures, target and actual suction pressures, irrigation volume	Wave	40 ms
Laparoscopic light source	Status (on/off), actual light intensity	Wave	40 ms
Surgical motor	Actual motor speed, maximum motor speed, upper motor speed	Wave	40 ms

#### System requirements and characteristics

The data recording system was designed to meet certain criteria and fulfil special technical requirements that are demanding for effective and safe data collection inside the OR. Firstly, the system could not disrupt the workflow of the surgical procedure. This was achieved by transmitting signals from the medical devices via an integrated Ethernet connection to a technical room outside the operating theatre. Secondly, the system could not interfere with the functionality of the medical devices. Indeed, the software for data exchange was developed according to the communication protocol provided by the manufacture of each device. Thirdly, automatic discovery of the start/end of the surgery to allow dynamic recording was necessary. Accordingly, connecting and disconnecting the patient to the anaesthesia machine and patient monitor were considered as the start and the end of the surgery. Finally, synchronous and structured recording of different data streams was required to enable valid correlation analyses.

**Figure 8:**
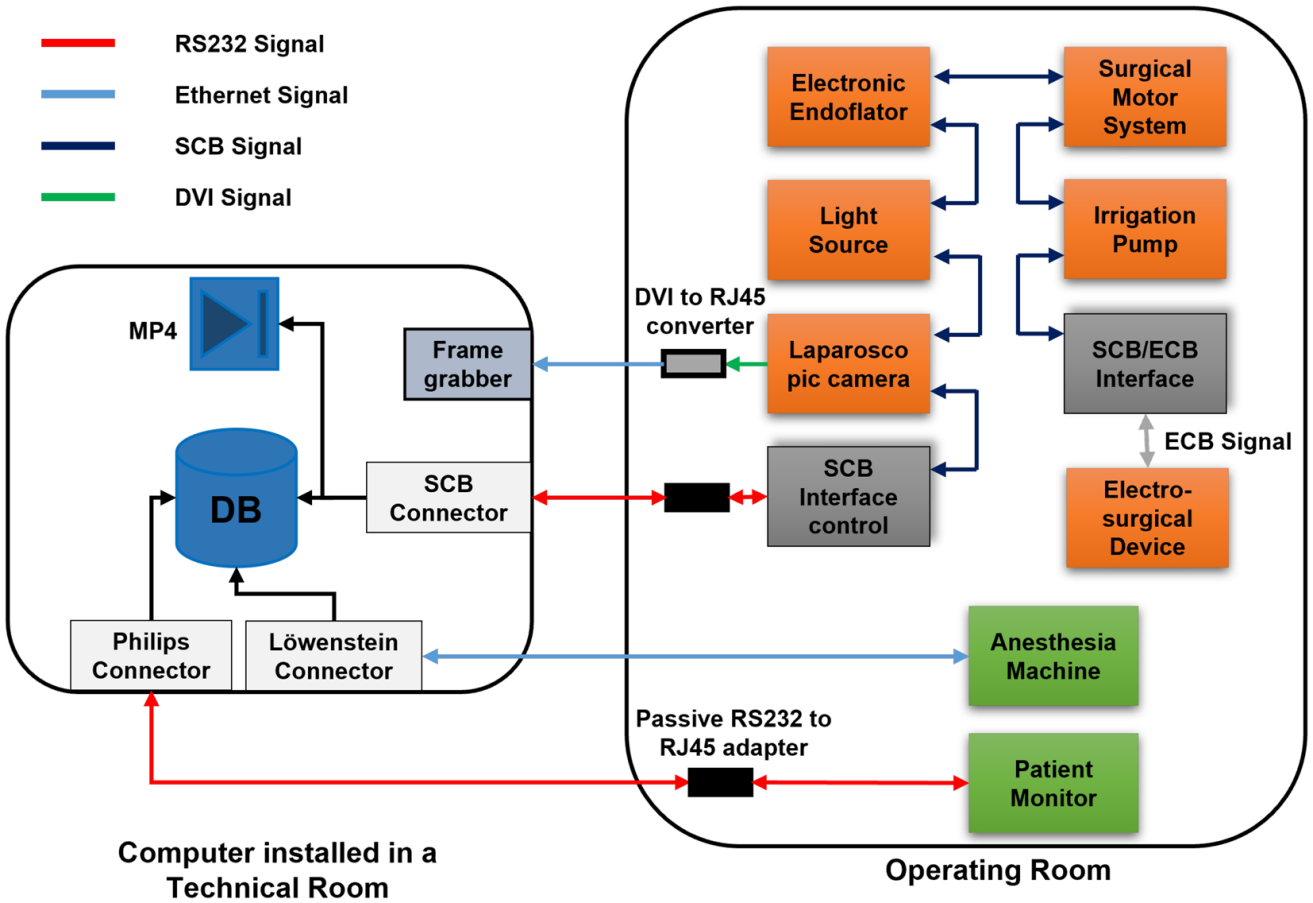
Schematic representative of the data recording system architecture. Philips Connecter, Löwenstein Connecter, and SCB Connector are the software for communicating with the patient monitor, anaesthesia machine, and surgical devices, respectively.

#### Hardware description

The data recording was performed in the integrated operating room OR1 FUSION (provided by KARL STORZ SE & Co.KG, Tuttlingen, Germany). This integrated OR facilitates interconnectivity of STORZ surgical devices (see Table 3 for complete list of included devices) via Storz Communication Bus (SCB). The electrosurgical unit (provided by ERBE Elektromedizin GmbH) had its own ERBE Communication Bus (ECB). However, a connection between the ERBE device and STORZ devices was possible via an SCB/ECB interface. By using an SCB interface control device connected to the SCB inside the OR, data from surgical devices was acquired via an RS232 serial connection interface. On the other hand, data from anaesthesia machine and patient monitor was recorded via an RS232 serial connection and RJ45 Ethernet connection interfaces, respectively.

The operating room had an integrated Ethernet connection to a technical room located within the surgical department. So, data was transferred via Ethernet connections to the technical room to maintain the surgical workflow not affected. Therefore, passive RS232-to-RJ45 adapters were required to transfer data from anaesthesia machine and the SCB control interface. Additionally, an active DVI-to-RJ45 converter was utilised to transfer video signal. The computer used for capturing data had an Intel$\circledR$ Core i7-2600 CPU, 8 GB RAM, and 1.81 TB free hard disk space. The computer was also equipped with two RS232 interfaces, an Ethernet interface, and a frame grabber.

#### Software description

The data recording software consists of three programmes written in C# and C++. The first programme, termed SCB connector, communicates with the surgical devices and writes medical data into a database. Additionally, SCB connector writes laparoscopic videos into mp4 files. The surgical data is sent automatically by the devices but without a timestamp. To synchronise data with acquired video frames, the SCB connector writes the frame number to the database table. The laparoscopic videos were acquired at 25 frames-per-second (fps). The second programme, Löwenstein connector, communicates with the anaesthesia machine and writes the received data into five database tables. These five tables include the static data, the ventilation and device settings, the monitoring values, active alarms, and the real-time waves. Table 4 defines the different types of data received from the anaesthesia machine. All data telegrams received from the anaesthesia machine have a timestamp. The local computer time was also saved into the database and used later as a reference to synchronise with other sources. The third programme, Philips connector, communicates with the patient monitor and writes numeric data and waves into separate database tables. The Philips connector allowed target signals to be requested from the device. The data was received at high sampling frequencies (ranging between 25 Hz to 500 Hz) from several devices simultaneously. Therefore, raw data was saved by the recording software without any pre-processing to avoid data loss. Figure 8 shows the connections of medical devices for recording data during laparoscopic procedures.

The software allowed automatic configuration of data sources to allow dynamic recording. If the patient was disconnected from the anaesthesia machine then reconnected, the system was configured to start new recording. However, the data of every connection was saved into separate files.

#### Data Pre-processing and Synchronisation

It is possible for the raw data to include missing or incorrect values. This can be caused by the data sources (i.e., medical devices), the data transmission, or the data recording software. Hence, data pre-processing is crucial to ensure consistency between data sent by varied sources. For the anaesthesiology data, every data message sent by the anaesthesia machine had a timestamp. This device-related timestamp was utilised as a criteria to detect missing data and correct incidences of incorrect data sequencing of received messages caused by processing of another response like ventilation settings. Missing data samples were added to the pre-processed data as Nan values. Similarly, data messages received from the patient monitor were also checked.

A deviation in the timestamp sent by the Philips monitor was observed. The Philips monitor sends two different timestamps. The first timestamp, absolute timestamp, has a resolution of 1 s and represent the local device time. The second timestamp, relative timestamp, has a higher resolution of 125 µs. The relation between absolute and relative timestamps can be estimated by requesting a specific data telegram from the monitor, called an MDS telegram. This telegram contains information about the software and hardware configuration of the monitor and the relative and absolute timestamps. Therefore, the MDS was requested at different times during the recording process, and the deviations between the two timestamps were estimated. The estimated deviation between the absolute and relative timestamps was about 1 and 5 s for 30 min and 3 h recording period, respectively. Moreover, the relation between the error and the elapsed duration was linear. A correction factor was calculated as:

1 $$\begin{aligned} CF = \frac{{\Delta }t_{abs}-{\Delta }t_{rel}}{{\Delta }t_{abs}} \end{aligned}$$

where *CF* is the correction factor, $\Delta t_{\textrm{abs}}$ is the elapsed absolute duration, $\Delta t_{\textrm{rel}}$ is the elapsed relative duration. The *CF* was then added to every relative time clock to correct the error in clock skews.

Numerical and wave data received from the anaesthesia machine or the patient monitor were synchronised with each other using the device-related timestamp. Conversely, data received from the surgical devices were sent without any timestamp. Hence, the laparoscopic video frame number was written to the database table and used to synchronise surgical data. To synchronise all signals from these different-vendor devices, the local computer time was used. A link between the local pc time and the device-related timestamps was determined based on the size of exchanged telegrams (request/response telegrams) and the data transfer speed.

### Data analysis

The abdomen of patients undergoing laparoscopic surgery is insufflated with Carbon Dioxide (CO$_{2}$) to create a sufficient working space for the surgeon. Consequently, the intra-abdominal pressure increases and thus forms a counter-pressure against the ventilator driving pressure. Compensating the effect of the increased IAP contradicts the target of the anaesthesiologist to ventilate the patient at low pressures. Hence, analysing the real-time relationship between the IAP and corresponding changes to respiratory mechanics represents an important aspect to enhance patient safety inside the OR. In this context, data of included subjects were processed and analysed with the focus on studying the correlation between the IAP and lung mechanics (dynamic lung compliance $(\hbox {C}_{\textrm{dyn}})$, peak airway pressure (PIP), and tidal volume $(\hbox {V}_{\textrm{T}}))$.

#### Patients and data

Data from nineteen female subjects who underwent laparoscopic gynaecology were included in this study. Eight subjects were ventilated with intermittent mandatory ventilation (IMV) mode, a volume-controlled ventilation (VCV) mode, while eleven subjects were treated with pressure-controlled ventilation (PCV). Settings of ventilation and the target intra-abdominal pressures utilised during the surgery for all subjects are shown in Table 5 and Table 6. The types of gynaecological procedures of all subjects are listed in Table 7. The measurements comply with all the relevant national regulations, and institutional policies and were performed in accordance with the tenets of the Helsinki Declaration. The Ethics Committee of Furtwangen University granted approval for the collection and use of the clinical data analysed in this study (application Nr. 19 -0306LEKHFU). Informed consent was obtained from all participants by the Anaesthesiologist.

**Table 5:** Ventilation settings and target IAP for volume-controlled ventilated patients.

Subject	Ventilation Settings					Target IAP [mmHg]
	Ventilation mode	Target tidal volume $(\hbox {V}_{\textrm{T,target}})$ [ml]	Respiration rate [1/min]	Inspiration/ Expiration ratio (I:E ratio)	PEEP levels [mbar]	
1	IMV	440 | 470	12 | 10 | 12 | 10 | 12	0.667 | 0.714	7	14
2	IMV	400 | 380	10 | 9 | 10 | 12 |14 |11	0.714	5 | 6 | 5	14
3	IMV	500 | 480	12 | 11 | 10 | 13 | 9	0.667 | 0.520	5	13
4	IMV	500	12 | 10 | 12 | 14 | 10	0.588 | 0.769 | 0.909	5	15
5	IMV	450	14 | 16	0.667	12	14
6	IMV	500 | 480 | 470 | 510 | 520 | 540 | 510 | 520 | 540 | 500	10 | 9 | 10 | 9 | 10 | 8 | 10 | 9 | 8 | 9 | 10 | 11 | 12 | 11 | 12 | 14 | 13 | 12 | 13	0.667 | 0.556 | 0.526 | 0.5 | 0.667 | 0.5 | 0.769 | 0.625 | 0.588 | 0.625 | 0.667 | 0.714 | 0.769 | 0.833 | 1.00	5	12 | 19 | 13
7	IMV	500 | 550 | 560	12 | 11 | 12	0.667	5	13 | 19 | 12
8	IMV	450 | 400 | 420	10 | 9 | 11 | 13 | 14	0.667 | 0.769	8	11 | 15

IMV refers to intermittent mandatory ventilation mode (a VCV mode).

**Table 6:** Ventilation settings and target IAP for pressure-controlled ventilated patients.

Subject	Ventilation Settings					Target IAP [mmHg]
	Ventilation mode	$\hbox {P}_{\textrm{ins,max}}$ [mbar]	Respiration rate [1/min]	Inspiration/ Expiration ratio (I:E ratio)	PEEP levels [mbar]	
9	PCV	24 | 29 | 33 | 30 | 20 | 18	12 | 16 | 14 | 16 | 18 | 16	0.667 | 0.5	10 | 12 | 8 | 12 | 10 | 12 | 10	14
10	PCV	18 | 20 | 19 | 30 | 27 | 32 | 34	12 | 11 | 13 | 12	0.667 | 1.00	6 | 5	14
11	PCV	15 | 20 | 22 | 35	12 | 10 | 8 | 9 | 11 | 13	0.667	5 | 8 | 9	12
12	PCV	18 | 16 | 21 | 28 | 30 | 27 | 28 | 24	12 | 11	0.667	5	14
13	PCV	13 | 15 | 14 | 15 | 16 | 17 | 19 | 20 | 22 | 24 | 22 | 24 | 23 | 21 | 23 | 24 | 20 | 21 | 20 | 18	12 | 11 | 12 | 14 | 13 | 15 | 16 | 14 | 13 | 12 | 11 | 12 | 13 | 14 | 12	0.667	5	12
14	PCV	15 | 14 | 16 | 15 | 20 | 22 | 24 | 27 | 28 | 12	8 | 9 | 8 | 9 | 8 | 9 | 11 | 12 | 14 | 10 | 13	0.667 | 1.00 | 0.714 | 0.667	5 | 8 | 5	12 | 14
15	PCV	14 | 16 | 19 | 22 | 24 | 25 | 26 | 25 | 24 | 23 | 22 | 18 | 19	12 | 14 | 13 | 12	0.667 | 0.769 | 0.667	8	12 | 22 | 11
16	PCV	12 | 13 | 14 | 16 | 15 | 16 | 17 | 19 | 17	10 | 11 | 13 | 14 | 16 | 17 | 16 | 15	0.667 | 1.00	5 | 7	12
17	PCV	18 | 15 | 12 | 14 | 17 | 18 | 15 | 13 | 15 | 17 | 18 | 20 | 17 | 16 | 17	9 | 8 | 10 | 9 | 11 | 13 | 14 | 15 | 11 | 12	0.667	6	12 | 14 | 13
18	PCV	17 | 18 | 19 | 17 | 20 | 22 | 21 | 20 | 22 | 23 | 24 | 23 | 24 | 20	14 | 11 | 9 | 10 | 11 | 12	0.667	6	12
19	PCV	16 | 18 | 17 | 20 | 22 | 24 | 23 | 25 | 23 | 14 | 18 | 19	15 | 9 | 8 | 9 | 11 | 14 | 13 | 14 | 11 | 13 | 14 | 13 | 14 | 16 | 15 | 14 | 13 | 10 | 8	0.667	6 | 8 | 7 | 6	12

PCV refers to pressure-controlled ventilation mode. ${P_{ins,max}}$ refers to the pre-set inspiration pressure.

**Table 7:** Type of surgical procedure for all subjects.

Subject	Type of surgery
1	Laparoscopic hysterectomy
2	Laparoscopic hysterectomy
3	Adnexectomy
4	Endometriosis
5	Laparoscopic hysterectomy
6	Laparoscopic hysterectomy
7	Laparoscopic Dermoid cyst excision
8	Laparoscopic sacropexy
9	Ectopic pregnancy
10	Hysterectomy + Adnexectomy both sides
11	Laparoscopic hysterectomy
12	Adnexectomy
13	Laparoscopic hysterectomy
14	Laparoscopic hysterectomy
15	Laparoscopic cyst excision
16	Laparoscopic sacropexy
17	Laparoscopic hysterectomy
18	Laparoscopic hysterectomy
19	Laparoscopic hysterectomy

#### Data processing

Signal filtering: Intra-abdominal pressure signals were acquired at 25 Hz. These signals were filtered using a low-pass Finite Impulse Response (FIR) filter prior to analysing the correlation with the respiratory mechanics values, which was the same as implemented in previous studies^34,35^. The FIR filter had a passband frequency of 40 mHz with attenuation of 0.5 dB. The stopband frequency of 40 mHz with attenuation of 50 dB was chosen. The FIR filter introduced a delay, that is constant at all frequencies, to the filtered IAP signal. This delay was calculated and compensated by shifting the filtered IAP in time to ensure alignment with other signals.

Determination of respiration parameters: The *PIP*, ${V_{T}}$, and *PEEP* were extracted from the respiration waves. The inspiration and expiration phases of every breath cycle were determined from the respiratory flow signal. The *PEEP* for every cycle was specified. The *PIP* and ${V_{T}}$ were also detected from the airway pressure and respiratory volume curves. The dynamic lung compliance was then calculated using

2 $$\begin{aligned} C_{dyn} = \frac{V_{T}}{PIP-PEEP} \end{aligned}$$

where ${C_{dyn}}$ is the dynamic lung compliance, ${V_{T}}$ is the tidal volume, *PIP* is the peak airway pressure, and *PEEP* is the positive end expiration pressure.

The pre-set values of inspiration pressure (${P_{ins,max}}$), target tidal volume $(\hbox {V}_{\textrm{T,target}})$, respiration rate (RR), inspiration/expiration ratio (I:E ratio), and *PEEP* were required for the statistical analysis. However, Ventilation settings were acquired every 60 s, compared to 20 ms sampling rate of the respiratory waves. Therefore, these pre-set values were interpolated to sampling frequency equivalent to the actual respiration rate.

#### Statistical study

The relationships between the IAP and ${C_{dyn}}$, *PIP* and ${V_{T}}$ were assessed by linear regression and Pearson’s correlation coefficient (r) for every subject. A multiple/multivariate linear regression (MLR) analysis was performed in order to consider alterations in ventilation settings during the surgery. Here, all relevant ventilation settings (respiration rate, I:E ratio, *PEEP*, and ${V_{T,target}}$ or ${P_{ins,max}}$ according to ventilation mode) were included in the correlation. All performed correlations are listed in Table 8.

**Table 8:** The analysed linear regression correlations, where *PIP* is the peak airway pressure, ${C_{dyn}}$ is the dynamic lung compliance, RR is the respiration rate, I:E ratio is the inspiration/expiration ratio, *PEEP* is the positive end expiratory pressure, ${P_{ins,max}}$ is the pre-set inspiration pressure for PCV mode, $\hbox {V}_{\textrm{T,target}}$ is target tidal volume for VCV mode, and IAP is the intra-abdominal pressure.

Correlation	Description
C	PIP versus IAP $\hbox {C}_{\textrm{dyn}}$ versus IAP $\hbox {V}_{\textrm{T}}$ versus IAP
MLR	PIP versus IAP & RR & I:E ratio & PEEP & ${P_{ins,max}}/\hbox {V}_{\textrm{T,target}}\, \hbox {C}_{\textrm{dyn}}$ versus IAP & RR & I:E ratio & PEEP & ${P_{ins,max}}/\hbox {V}_{\textrm{T,target}}$ $\hbox {V}_{\textrm{T}}$ versus IAP & RR & I:E ratio & PEEP & ${P_{ins,max}}/\hbox {V}_{\textrm{T,target}}$

## Conclusion

To demonstrate the utility of a synchronised data recording system, an exemplary study was conducted that analysed the effect of intra-abdominal pressure on lung mechanics during laparoscopic surgeries. Statistical analysis demonstrated a strong correlation between the intra-abdominal pressure and the lung compliance of the patient during laparoscopy. Moreover, the results obtained demonstrate the potential of fusing and combining data from anaesthesiology and surgery to generate a comprehensive understanding of the situation inside the OR. Consequently, patient safety and surgical treatment can be optimised.

## Data Availability

The dataset used and analysed during the current study is not publicly available. However, data are available from the corresponding author upon reasonable request.
